# Abstract of Proceedings of the Buffalo Medical Association

**Published:** 1867-09

**Authors:** T. M. Johnson

**Affiliations:** Sec’y.


BUFFALO

Medical and Surgical Journal.

VOL. VII. SEPTEMBER, 1867. No. 2.

### Original Communications.

ART. I.—*Abstract of Proceedings of the Buffalo Medical Association.*

TUESDAY EVENING, August 6th, 1867.

The meeting was called to order at the usual hour by the Vice President, Dr. J. R. Lothrop. Members present—Drs. Lothrop, Strong, Wetmore, Abbott, Wyckoff, Gould, Nichell, Little, Potter, Lockwood, Jansen, Cronyn, Congar, Gay, Smith and Johnson.

The minutes of the last meeting were read and approved.

Drs. M. G. Potter and A. E. Mackay, were elected members of the Association.

The following gentlemen made application for membership:—Drs. C. F. A. Nichell, Conrad Diehl and B. H. Daggett.

Dissertations on designated subjects being in order, Dr. P. H. Strong presented the following dissertation:

*Typhoid Fever—Is it Arrestable? The Affirmative Presented.*

Mr. President and gentlemen of the Association:

The subject of *Typhoid Fever*, (so called), has filled a large place in the medical mind, and a large space in medical literature, and that for many years. Some of the best talent of the profession, especially during the present century, has been engaged in exploring the field, and in making record of its explorations and deductions, insomuch that it may almost seem a trite and well nigh exhausted theme, to dwell upon which, may be thought, if not presumptuous, at least profitless.

I have selected it as the theme for the evening, not to make an historical *resumé* of discarded or received theories and opinions upon it, not to describe its symptoms, nor to enter at length upon either its etiology, therapeutics or pathology. The subject, thus considered, is altogether too vast, and already too well elaborated to make it either necessary or excusable in this presence, and in our brief interviews to give the natural history of the subject. What I have proposed to myself is more a work of *reconciliation*, than aught else; by which I mean, to reconcile medicine with itself.

Fever, in the abstract, as to its nature and essence, has for centuries been an open question. The day has not yet dawned when we can fully solve the problem as to many of its types and forms. But it has seemed to me for years that the time has fully come when the typhoid affection, (which designation I prefer to that of typhoid fever,) should be eliminated from the category of *opprobria medicorum*, at least so far as pertains to its proximate cause, the order and relation of its essential and multiform phenomena, and its indications for treatment.

The tendency of the medical mind of the present day, it seems to me, is more and more strongly to the conviction that every form and phase of disease, howsoever insidious and occult, is the product of some lesion of structure. Not that we can always, with our present means of investigation, trace to its ultimate source and fix the exact point of departure from the normal state, either by unaided or *aided* sense. But the tendency is with ever increasing strength, not to rest short of *just that* goal. Even insanity instead of being regarded merely as a functional phenomenon, as formerly, is found to be in most cases, and believed to be in all, traceable to lesions of brain structure as its *causa sina qua. non.* Pyæmia, or any other form of blood poisoning, whether its assaults may be made upon the mysterious organization of the nerves and nerve centres, upon the hardly less intangible structure of the secernents and absorbents, or a combined assault upon both, cannot be allowed to be an exception to the rule. Change and modification of structure probably always exists, and must always be sought, if we would profitably study or successfully treat disease.

But, not to dwell upon generalities, what is the truth in regard to this point, as pertaining to the typhoid affection? According to *Louis'* post mortem investigations, while other lesions of various organs and tissues were not wanting—such as the mesenteric and mesocolic glands and the spleen, the mucous membrane of the cœcum and colon, of the stomach, pharynx and œsophagus, of the bronchial membrane, and the pulmonary structure, of the brain and its meninges, etc., etc. Yet all of these were ever varying in kind and degree, existing in some cases and non-existing in others, but originating consecutively or secondarily in all. While the *elliptical patches of Peyer* were in varying stages of disease in *every case*, without exception. This disease varied in intensity and in extent of disorganization from a point nearest the ileo-cœcal valve, where was uniformly found its maximum, upward one, two, three, four feet or more of the ileum. Near its lower terminus were found often more or less of sloughing of the glandular structures and of sub-mucus tissues, sometimes the unsloughed deposits of what is called the typhoid material in these parts; next, ulceration of the mucous membrane covering the patches; higher up, or in the direction of diminishing severity, softening and thickening of the patches from one to three lines in depth, and from a few lines to as many inches in surface measure; higher still, yet immediately contiguous, were found lesser stages of softening and thickening, merging to the simple stage of incipient inflammation. (I owe an apology for this very imperfect and almost unintelligible description of a pathological condition, to do justice to which, would require as many pages as my limits will allow me to devote lines.) These unique structural disorganizations and lesions were found so uniformly, (varying only in number and in degree as the history of the case was more or less protracted,) as to deserve and receive from *Louis* the designation of the "*Anatomical Characteristic.*"

Now the question is pertinent: What relation does this singular disease of these patches of glands bear to this affection? Opinions seem to differ upon this point. My time will only allow of my referring to those of Dr. Louis and Dr, Flint, the two highest authorities living or dead, as I regard them, upon this subject.

The question at issue is not so much one of fact, both agreeing as to the existence and the uniformity of the lesions, as of inference and deduction, or, as to what this lesion has to do with the disease under consideration. If I rightly understand Dr. Louis he regards the incipient disease of the patches as the initial point of departure from health, the primary lesion; or, in a word, at once the *beginning* and the *cause* of the fever. If I rightly apprehend Dr. Flint, while he admits that the affection of the agminated glands is a general, perhaps even *constant* accompaniment, it is *only* an *accompaniment* of the fever. In brief, it is one of its symptoms or incidents.

Inasmuch as precisely upon this point will hinge most of what I shall have to offer you, gentlemen, this evening, and in order that no injustice shall be done to my eminent authority, it is only fair to quote his words, to which I would now invite your careful attention.

Alluding to case under analysis, Louis says: "They (the symp- "toms) commenced at various periods of day, by chills or trem- "bling, or both, headache, universal feeling of lassitude, anorexia, "thirst, some pains in abdomen, and, in the majority of cases "liquid dejections supervened during the first twenty-four hours. "Heat followed the chills, and then recurred several days in suc- "cession in nearly all the subjects—after which the skin was con- "stantly more or less hot, and nearly always dry. These symp- "toms presented nothing in them peculiar to any disease, and "showed that the affection had its seat in the abdomen, and they "successively became more and more violent. A little earlier or "later, at different periods of the disease, other symptoms began "relating to the cerebral and abdominal functions, to the organs "of sense;" etc.

Again he says: "That in these cases, (alluding to cases in which "was found the characteristic lesion of the ileum,) the ileum must "be considered as having been the principal seat of the disease, "and source of the *principal* and *first* changes in the system of the "patient. Again, in this case, as in the others, the *first* symptoms "can be attributed to the commencing disease in the ileum."

Again—"Between the symptoms and lesions of which we are "treating, the relation seems to me to be not less evident than

"that which is obtained between those two orders of facts, as they "take place in other affections—in *pneumonia* for example."

Again—"I do not see how it is possible to doubt that the "period at which these lesions began was exactly the same as that "of the disease."

Again—"Facts show that almost all (if not *all)* the symptoms "observed in typhoid fever, and especially those that may be "called characteristic, *depend on* the peculiar morbid change in "the ileum."

Of other coexisting lesions, he says: "Every time that there "were complications, and one could by the aid of the symptoms "discover the periods at which the different lesions commenced, "that of the patches of the ileum was evidently the *first.*— "And as in nearly all the cases which died, the *first* symptoms were "connected with a lesion in the intestinal canal, we must conclude "that the time at which the alteration of the elliptical patches "commenced was the same as that of the disease, and *we must not* "*consider this lesion as one of the effects of the fever*, but that it forms "the *anatomical characteristic.*"

So far on the general subject. I will now collate for you a few of his remarks upon the relation of some of the individual symptoms to the primary lesions. Of the extreme lassitude and debility which ushers in and attends the disease Louis says: "But to what "cause shall we refer the extreme debility observed in the majority "of the cases? When it occurred at a late period of the disease "we could to a certain extent, explain it by the condition of the "organs, and the long disorder of the functions of the body, "although so great a prostration rarely occurs in the course of "other acute diseases. But in those cases in which there was "considerable debility from the commencement, (which was com- "mon,) we could not give this explanation. We could not attrib- "ute it to the diarrhœa which did not then exist, or was very "slight, nor to the abdominal pains, which were then not severe, "nor to the cephalalgia which was generally dull, and the severest "attacks of which in other acute diseases do not produce like "diminution of strength, nor to any appreciable lesion of the "brain, for the reason already given, nor to the state of the mu- "cous membrane of the stomach, which was healthy in many

"cases, where there was extreme prostration of strength, and "whose alterations moreover commenced at a somewhat remote "period after the disease began. It is, therefore, necessary in "order to explain this symptom, to recur to the special lesion "which was just beginning, as acting sympathetically upon the "brain, or still farther to the cause, whatever it may be, which "produces this lesion."

Of the *diarrhœa* Louis says: "The alteration of the patches was "doubtless the cause of the abundant alvine discharges."

Of the *delirium* he says; "As there was only one lesion which "was constant, and always the same in all subjects, viz: the alter- "ation of the elliptical patches, we must infer that it is in this last "lesion, and not in any other that we must look for the cause of "the delirium, and more especially of the somnolency."

Again—"Therefore we arrive rigorously at the conclusion, that "we must place the cause of the drowsiness in the peculiar altera- "tion of the patches, and this deduction is strictly true for all "the cases."

The existence of the pain in the right iliac region, whether without pressure, as in many cases, or by pressure in nearly all, is too palpable and too significant to require citations from Louis. And of the other symptoms, attending the development and progress of the disease, I will, without quoting, simply make the general remark that he traces them, one and all, to the same prolific source—the lighting up and progressive development of inflammation and its sequences in the elliptical patches.

And now, concerning the relation of some of the accompanying pathological lesions—to the primary affection—I will quote a few specifications as types of the whole. He says: "The disease in "the mesenteric glands corresponding to the ileum, comes in sup- "port of this opinion—this alteration which was severe, necessa- "rily having been *consequent* upon that of the patches."

Again—"It is certain that the increased size and rose color of "these (the mesenteric) glands could be attributed to *that cause* "*only which produces* so many other secondary lesions, viz: the "disease in the ileum. Thus it appears that the typhoid disease, "(in the ileum) seems to establish a marked predisposition to "morbid changes in the mesenteric glands, but not merely in those, "but in those of other regions, particularly of the neck."

Of the lesions found in the large intestines he says: "The red-"dening, softening and thickening of the mucous membrane of "the large intestines ought to be considered as *secondary* lesions, "or as consequent upon those of the ileum."

Of the brain and meninges he says: "The lesions of the brain "and its membranes, were generally very slight, and doubtless the "product of the last hours or days of life."

And so on, as to the other morbid appearances in organs and tissues, which time and purpose forbid to quote, I will only further give you in this connection his comprehensive and note-worthy conclusions, thus: "Whence we must conclude that it (the typhoid "affection) differs from others, not only by its seat and the char-"acter of its lesions, *but by an important peculiarity bestowed by it,* "*upon the different membranes and tissues of the body, which tend to* "*produce ulceration in them! So that in this respect the typhoid affec-*"*tion is to other acute diseases what phthisis is to chronic disease.*"

Of a case or two among his post mortem investigations, which, to a less careful and candid observer, might seem to conflict with some of his conclusions based upon the almost unvaryingly exten-sive and destructive lesions of the patches, Louis says: "It prob-"ably never happens that individuals who die of a disease, the "seat of which is well determined, are free from lesions in other "organs besides that primarily affected; at least I have never met "with any example. But in the larger number of these cases, "the disorder of the organ is so great that death is explained "easily by it, and we can to a certain extent neglect the consider-"ation of the secondary lesions. But in others the primitive "lesion is so slight, either from its commencement, or because it "has retrograded sometime before death, that death cannot be "explained without having recourse to accessory lesions, and we "cannot but admit that had it not been for the addition of these "to what previously existed, the patient would probably have "recovered from the primitive affection."

Let it be borne distinctly in mind, gentlemen, that these quota-tions from Louis (and they might be extended indefinitely) were not only *bed-side* but *dead-room* conclusions, predicated upon forty-eight cases of fatal typhoid affection, and most scrutinizing inqui-sition by the knife, into all the organs and tissues of the body, followed by patient comparison and exhaustive analysis.

Now, whatever may be the short-comings of the French medical man, and of Louis, the representative Frenchman, it must be conceded, I believe, that in the *dead-room*, he is a model. His pathological researches are so inquisitively and thoroughly made, so honestly, clearly and vividly recorded—his deductions and conclusions are so rigorous, fair and legitimate, so free from bias and preconception, that it is simply impossible to arise from the careful study of his work on this disease, without the ineradicable conviction that it is something reliable, *something to lean up against* in reference to the facts of the disease after death, and, for the most part, in reference to the inferences and deductions drawn from the order and succession of its varied phenomena during life, and their relation to the fatal issue.

What *were* his conclusions pertaining to what was revealed by the knife, after death, from typhoid affection, and its relation to the development of the disease during life? Briefly these, viz: That from some occult cause disease was kindled up in the Peyerian patches; that it was the initial departure from health, so far as known; that the first symptoms of febrile movement, the chills or trembling, lassitude, headache, anorexia, thirst, etc., were caused by this incipient inflammation; that these symptoms increased, and others supervened, *parri passu*, with the increase of this local affection, where begun, and its extension to other points; that the first symptoms are *entirely explicable* by this lighting up of disease in these peculiar structures; that the diarrhœa when commencing early in the disease (which was usually the fact,) was wholly referrible to this cause; that when the inflammation was sufficiently developed to cause pain, either without pressure or *by* it, the seat of such pain pointed unerringly and almost without exception to the region of these patches; that the remarkable and continuous heat of surface could only be explained by this structural lesion; that the early accompanying disease of mesenteric glands in immediate proximity was secondary to and caused by the primary lesion; that the later symptoms—the meteorism, the drowsiness, the delirium, the affection of the special senses, the lenticular rose eruption, the sudamina, the ulceration of the pharynx and œsophagus, when existing, and all the other symptoms which are manifested sooner or later, as the disease advances, are all and singular, the sequence of the advancing and deepening disease of the patches as it passes on to and through its stages of softening, deposition of material, sloughing, absorption, ulceration and perforation, operating through the glandular sanguiferous and nervous systems as a blood poison or ferment, and thus exciting morbid sympathies and developing morbid products with more than geometrical progression. In a word, that all the symptoms manifested during life, and all the revelations of diseased structure after death, have for their initiative, their *fons et origo*, the disease kindled in one or more points in the Peyerian patches, and spreading therefrom. They are all secondary as to time, and sequent as to relation to the primary affection. If I mistake not Louis' opinion is that the affection is primarily a specific inflammation, made such by the nature of the occult agency acting upon these glands or by their peculiar anatomical structure, and their mysterious functions and relations to the mesenteric and mesocolic systems, and through *them* upon the entire organism. So much, and as concisely as possible, for the views of Dr. Louis and those who hold with him.

Dr. Flint, (and others,) whom my space forbids me to quote, as before intimated, while he recognizes the constancy of the Peyerian lesions, and, it may be, their gravity and significance, yet regards the disease as an essential fever, and these lesions as playing a part, perhaps a *leading* part in its symptomatology; or in other words, that the typhoid disease is a blood disease, primarily and essentially, and that the local affection of the patches is only one of its developments or symptoms. I hope and believe that this statement does no injustice to his views without lengthy citations. How he or any one who holds with him, can reconcile the facts that the Peyerian affection is an invariably constant element, while all the other symptoms and lesions are ever shifting and variable in kind and degree, and more especially the stubborn and conspicuous facts, that the very earliest and first symptoms during life, and the lesions after death *point to* and are *solvable by* this affection, while all the others are more or less secondary and consecutive. I repeat, how *any one* can make these facts consist with classing them all alike, as symptoms merely, I will not undertake to say.

My object at present lies not so much in reconciling one authority with another, as in reconciling each with himself. One authority regards it as an essential fever, and treats it as such, and so far perhaps has the merit of consistency. But even *he* admits the affection of the patches is an important element in the fever, and its lesions, though symptomatic, are the gravest and most perilous; admits even that it is the first in the order of succession, and that the nature of the disease is inflammatory, specific and peculiar, if you please, but still inflammation, of a peculiar structure, having a peculiar function and extensive sympathetic relations to and affinities with the whole system.

Now, the query that has long dwelt unsolved in my own mind, and now finds expression, is, why this local inflammation playing concededly so very important and dangerous a part in the fever, as it does, *is not entitled to be treated* as a local inflammation? Inflammation, localized in any other organ or tissue, is *treated* as such — locally, if accessible, and systemically, if needful. Who thinks, for example, of treating inflammation of the brain, or its membranes, or of the stomach, or peritoneum, especially if it is possible to localize it, without efficient *local* treatment? Even when either of these structures or organs become affected consecutively in *this fever*, who fails—author or practitioner—to resort to local means for its abatement? And yet, we meet with the strange inconsistency of allowing *this* local affection, clearly of inflammatory nature in the first instance, confessedly the chief, if not the *only* source of fatal lesions in many cases. and of danger in all, to go unchecked and ignored so far as adapting efficient local measures to it, is concerned.

I do not forget that we are enjoined to give opium to relieve abdominal pain, and to apply emollient and other applications—possibly mustard—but all mainly with the idea of relieving suffering. Do we treat meningitis or pleuritis or stomatitis, with such mild means? To ask it is to answer it.

*Theoretically*, then, it seems unphilosophical and indefensible, in one who only regards the disease as merely a symptom of the fever, and yet admits it to be chief source and cause of danger, to adopt no local means to these local developments of disease and local sources of peril.

But of how much greater urgency is it to rescue from his inconsistency the author who views it as the *initial* point of morbid action, the *fons et origo* of the typhoid affection—the main, it may almost be said, the *only* cause of fatality—certainly the chief cause of the prostration and of the tedious convalescence so common in the disease. For such an one to utterly ignore this local source and cause of danger and death, is passing strange! And yet it is precisely this which Louis does, in his record of treatment, with hardly an exception. I can scarcely call to mind a single instance in which he adapted any local measures to eradicate or obviate the local disease of the patches. So far as *treatment* could indicate it, the idea of disease of the elliptical patches was the merest chimera concievable. True, he applies leeches, but he finds no good place for them except about the head to obviate secondary brain complication! True, too, he resorts to the epispastic, but only upon the nuchæ, or the arm, or leg, can a suitable place be found, and then late in the disease, and with a vague idea of modifying some secondary symptom or lesion. The true seat and origin of disease, as he regards it, and as *he abundantly demonstrates* it by his post mortem researches, is wholly, systematically left out of view, until, forsooth, death supervened, and then we have a revelation of disorganization and destruction which he portrays for us with photographic vividness! No other adequate cause of fatality than the destructive lesions of the patches, and yet no attention, absolutely *none*, paid to them while under treatment! 'Tis strange, passing strange!

Theoretically then, gentlemen, this mode of treating the typhoid affection seems inconsistent and untenable. I shall relieve your patient attention when I have presented a few facts bearing upon the question at issue, and confirmatory of my position, and a few deductions and observations founded upon them.

Case 1—Called December 1st, 1865, to W. D., South Division street, male, aged 17, well developed, generally healthy and vigorous. He had complained for two or three days of headache, lassitude, etc. Found him unable to sit up from debility and dizziness; had had a partial chill and trembling; now had severe headache, though inclining to sleep, anorexia, thirst, heat of surface, pulse about 95, slight inclination to diarrhœa, pain in abdomen, slight tenderness in right iliac region. Prescribed spts. nit. dulc. and quieting opiate. Diagnosis as to its being febricula or typhoid fever, undetermined.

Dec. 2d.—Found symptoms much the same; pulse about 100; tenderness in right iliac increased; several dejections from bowels; heat of surface and debility more marked; somnolency, anorexia, thirst considerable. Diagnosis still undetermined. Prescribed calomel grs. v, fiat pulv. ij, one to be taken three hours after the first, to be followed by seidlitz powder, dejection not being secured without; succeded by opiate.

Dec. 3.—Bowels had been acted on, with characteristic discharges. Found pulse about 100; heat, thirst, sleepiness, prostration, pain on pressure in right iliac continuing much the same, inclining to delirium and slight tympany. Exact diagnosis still in abeyance. Continued treatment.

Dec. 4.—Found pulse about 110; other symptoms much the same, though slightly aggravated; epistaxis added. The diarrhœa and pain and pressure in the right iliac continuing, became satisfied of its continuous typhoid character. Prescribed opiate, spts. nit. dulc., epispastic 4x4 in. over region of right iliac.

Dec. 5.—Found pulse about 110, still inclining to somnolence and delirium; other symptoms not materially different; blister had just drawn. Diagnosis confirmed. Continued treatment.

Dec. 6.—Found him more easily aroused and clearer in his mind. Pulse 105; diarrhœa, thirst, heat, abdominal pain, slightly less. Continued treatment.

Dec. 7.—Found all symptoms slightly abated. Pulse about 95; diarrhœa under control; mind clearer; more tolerance of pressure in right iliac ; debility and heat less marked; slight desire for food. Continued treatment superadding two grs. of sulph. quinia every four hours.

Dec. 8.—Symptoms not greatly less, but all in that direction. Pressure over Peyerian patches better borne, though still slightly painful. Continued treatment.

Dec. 9.—Symptoms improved in every respect; culmination apparently reached.

Dec. 11.—Convalescence has continued without interruption. Local symptoms in right iliac hardly noticeable, and so on (not to dwell) gradually though steadily till well. Attendance discontinued on 16th.

My impression from the *ensemble* of the symptoms, was and is, that it would have been a case of grave, protracted, and perhaps fatal typhoid affection, but for the abortive treatment by the epispastic, etc.

Case 2—Called September 29, 1866, to Mrs. C. P., Perry street, aged about 24, spare, though vigorous. She had had prodromous symptoms, ending in chill, for three or four days previous, keeping about house. I found pulse about 85; anorexia complete; tongue slightly furred and dryish; frontal headache severe and continuous; thirst and lassitude extreme; abdominal pain severest on pressure over right iliac region; manifested great anxiety, etc. Prescribed quieting opiate and spts, nit. dulc. every four hours.

Sept. 30.—Symptoms not greatly varied, though all in the direction of increase; heat of surface marked and unintermitted; bowels inclining to looseness. Full diagnosis in abeyance. Continued treatment.

Oct. 1.—Symptoms slightly aggravated; pulse about 95; headache severe; prostration extreme, inclining to faintness; great intolerance to pressure over right iliac ; slight diarrhœa. Prescribed calomel grs. iii, in three hours the same; movement of bowels to be followed by opiate, every four hours.

Oct. 2.—Symptoms aggravating; pulse 110; anxiety changed to hebetude, though conscious of headache and abdominal pain, especially on pressure in right iliac. Becoming confirmed in opinion that she had incipient typhoid affection, prescribed continued treatment, and added epispastic over right iliac.

Oct. 3.—Symptoms not greatly changed, though in the direction of aggravation. Pulse about 105; somnolency and debility increasing; blister had not fully drawn, so continued it and other treatment.

Oct. 4.—Blister complete; symptoms not very different, though with less of abdominal pain and fewer dejections. Continued treatment.

Oct. 5.—Symptoms continued much as before, with slight increase of drowsiness. Continued treatment.

Oct. 6.—Symptoms continuing and of deepening tendency, from increased hebetude; complained less of pain of head and abdomen; still, as blistered surface would now admit of pressure upon it, found considerable tenderness over right iliac with some meteorism. Satisfied that the disease of the patches, though checked, was not adequately subdued, I direeted repetition of blister and continued treatment.

Oct. 7.—Blister well drawn; symptoms not greatly changed—certainly not aggravated; pulse four or five less.

Oct. 8.—Symptoms generally mitigated; pulse 100; heat and thirst less; diarrhœa checked; hebetude somewhat abated; pain not mentioned; more competent and disposed to muscular exertion. Continued treatment.

Oct. 9.—Symptoms still in the direction of improvement. As the seat of the blister would now admit of pressure, found less of tenderness and meteorism. In brief, the disease had culminated, and convalescence was established, and continued very slowly, though very steadily to complete restoration. Attendance was continued five or six days from this date, but with no anxiety or doubt as to the result, so that giving the daily record is superfluous.

Case 3.—Was called November 3, 1866, to Mrs. V. C. N., Tenth street, aged about 38, of fleshy, full habit, and general good health. She had been ill a few days, though about the house. Found her unable to sit up from debility and dizziness. Pulse about 98; had vomited; had headache, anorexia, thirst, abdominal pain, with great intolerance of pressure in right iliac region, great mental agitation, several liquid dejections, etc., etc.

But, in order to abbreviate my paper, rather than give the daily record, I will concisely say, that the history of this case and of its treatment was nearly a repetition of case two, excepting that I applied the epispastic some day and a half earlier, and had a corresponding abatement of urgent symptoms and culmination of the disease, and also obviated the necessity of the second epispastic. If my diagnosis in these cases is open to question, I can only say that they (especially Nos. 1 and 2) lacked few, if any, of the characteristic symptoms of the *early* stages of the disease. Of course the lenticular rose spots, the sudamina, the severe brain and nerve symptoms, etc., were not present, because, according to

Louis and Flint, they are incident to later stages of the affection. The eruption rarely appearing sooner than the second week, and the sudamina and severe brain and other complications at a still later period, and all of these and all of the other symptoms are of ever varying degree, even when existing, which none of them always are. They are all secondary to and symptomatic of, the one primary affection, requiring the kindling of morbid sympathies and the absorption of morbid products of the diseased patches, with the accompanying highly fermentative febrile movement for their development and dissemination.

I do not need to be reminded, gentlemen, that neither one swallow, nor three swallows makes a summer. Neither can three cases settle a disputed question of treatment. I only claim that in these cases the disease, whatever it was, was *aborted* by the thorough application of epispastics, inasmuch as the affection showed not the slightest indication of abatement until this measure was adopted, but a marked abatement immediately thereupon.

To my own mind they serve as strongly confirmatory of Dr. Louis' view of the affection, that it is primarily and essentially a disease of the Peyerian patches. But even upon the supposition that this affection is only one of the symptoms of an essential fever, yet, with the concession that it is one of the *earliest* symptoms, and always indicates the seat of the gravest lesion, and therefore the source of the greatest peril, it seems to me to clearly fulfill the most urgent indication, and by consequence to be the most rational and consistent treatment.

Is the objection raised that it sometimes occurs in this affection that the indication of pain in the right iliac, even by pressure, is slightly or wholly wanting; yet, according to Louis, predicating the affection to be typhoid fever, the incipient disease is none the less *there*, though latent, masked and undemonstrative. And *if* there, I would like to make Louis consistent with himself, by treating it according to its nature and seat.

It perhaps ought to be said, in extenuation of the practice of Louis and others, that, being cases in hospital, and therefore generally coming in hand with the disease fully established and often well advanced, recourse could not be had to abortive treatment with hope of success. But in this class of cases, with the evidence which post mortem research has furnished, that while the affection of the glands first involved may have passed on to the stage of deposition of typhoid material, or sloughing, or ulceration, yet other portions of the patches in immediate proximity, are in varying stages of inflammation, from the most incipient to the most advanced, it seems still most obviously indicated, that the *extension* of the disorganizing process should be obviated or stopped; and that local treatment by persistent, and if need be repeated, blisters, or these preceded by local leeching will effect just *that*, I firmly believe, and therefore earnestly insist.

Moreover I am very far from conceding that these measures are powerless, or, are not of great potency in modifying and limiting the later stages of this destructive process, viz: softening, sloughing and ulceration. Whatever theory we may adopt in reference to the nature of these processes, while I have no experience bearing upon the point, I believe future observations will establish this treatment, as just what is required, and therefore our most available resource in limiting and controlling these processes, and thus obviating that common and inevitably fatal termination, perforation of the peritoneal coat of the intestine.

I speak above of local leeching, not from observation, but inferentially, and from analogy. To my own mind there is no internal organ or tissue in the whole system that responds more if *so* readily and agreeably to prompt and thorough surface treatment in immediate contiguity, as do the Peyerian structures. In my judgment leeches are decidedly indicated, when for any reason the epispastics are inapplicable or ineligible. It would probably be wise to precede the latter by the former, in any case where there is the least doubt that the epispastic, first or second, would be effectual. In my hands the single epispastic, or its repetition, has proved all that could be desired. But analogically there can be little doubt that in any urgent case leeching is strongly indicated, nor that it might be frequently expected to preclude the necessity of the epispastic. If used at all, of course it must precede the latter.

I would make no issue, gentlemen, upon the received views of authorities, as to the indications for treatment of fevers or other affections, when there are no obvious and palpable causes for the febrile movement. Being in the present state of knowledge purely systemic affections, involving no one organ or tissue more than another, we are shut up to the necessity, perhaps, of treating them *systemically.*

But I humbly submit that, from the light thrown upon our pathway, by the symptoms during life, and preëminently by the scalpel, after death, the affection under review, should no longer be left in that category; to do so, in this particular, it seems to me, is to make questionable the claim for our science and art, to the title of *rational* medicine. Theoretically it is unreasonable and illogical. And the logic of events and of facts speak equally, if not even more strongly, against the received view, and in favor of a reform or an *advance.*

A choice fruit-tree is found shriveling in its foliage and blasting in its fruitage. Does the expert horticulturist spend the least time or thought in directly treating either leaves or fruit? Does he not rather search out for their annihilation, the canker-worms at its root, or apply his remedies to the ill-adapted or pernicious soil? Or, as more closely illustrative: In a long standing case of disease of the os or cervix uteri, do we call it good practice *now*, to waste time and energy in administering directly or systemically to the cephalalgia, or other neuralgia, or to the heart palpitation, or to the general debility and incapacity for exertion? True, the time and within the memory of most of us, when, from our ignorance of its true pathology, and in the absence of the speculum, we spent many months, and it may be years, in ministering to the symptoms and incidents of the disease as they were manifested in distant organs and functions, and ever with unsatisfactory results, either to our patients or ourselves. But now, the direct application of the *caustic*, has changed all that, and our symptomatology and pathology are *reconciled* to our therapy, aud the *trio* instead of being discordant as formerly, are brought into unison and harmony, and thereby are a credit and an honor to legitimate medicine. Precisely this, and nothing more, it seems to me is demanded in regard to the typhoid affection.

This done, and while I have no aspirations for the honors of a prophet or the son of a prophet, me thinks there cannot be great presumption in predicting, that ere long, protracted, and especially *fatal* typhoid affection, shall be known only as a thing of history.

Dr. Gay said the paper read by Dr. S. was elaborate, well written, and pleased him much, but it had failed to convince him (Dr. G.) that typhoid fever was abortable. The first question presented to our minds in the dissertation is that of the controversy between the solidists and humoralists. The Doctor, it seems, is a solidist, and a disciple of Louis, the primary seat of the typhoid fever is in the glands of Peyer—the mucous membrane inflames and ulcerates; in short, the fever is at first an inflammation, and if promptly met by vesicants, etc., the ordinary phenomena which occur in developed fever will not appear, but the disease will be aborted. The Doctor's reasoning is deductive, and if his premises were well established, his conclusions would have some show of truthfulness. But I assume that typhoid fever is not primarily a lesion of the glands of Peyer ; that lesion of these glands is an intercurrent affection, that in obedience to certain immutable laws pertaining to and inseparable from the fever, the mucous membrane, becomes the seat of local inflammation, as in the exanthemata the skin becomes the seat of local inflammation. Scarlatina and variola are recognized diseases, even when there is no eruption; the eruption does not constitute the malady; it is but secondary or consequent upon, and is part of the disease only. The disease itself, is a blood poison, a general disease, whether it be scarlatina, measles, or typhoid fever. What the blood poison is I cannot say; occasionally we see a patient overwhelmed by the poison, and dying at once—post mortem showing no lesion of the intestines. By a process of catalysis, poison, acting upon the blood decomposes the blood, or the blood like any other compound body becomes decomposed without the agency of any poison; when in its decomposed state, from whatever cause, whether it be poison conveyed from without or otherwise, is it not a poison, and a sufficient poison in such abnormal state to account for the production and all the phenomena of fever—the mildness or intensity of the fever depending upon the amount or degree of such decomposition? When a patient is at once overwhelmed, post mortem reveals the blood entirely decomposed, and in a fluid condition; when the fever runs on a mild course the indications are that the blood undergoes only partial decomposition.

If the views of the humroal pathologists be correct, then we see the propriety of those expressions sometimes made use of with regard to typhoid fever, that it is or is not of the Peyerian type, and the position I assume to be true, that typhoid fever may or may not be complicated with ulceration of Peyer's glands.

To sum up the whole matter the Doctor, it seems to me, has placed, or rather misplaced, cause for effect, and vice versa, and although his paper is interesting and very instructive, he has clearly failed to make his point in the case.

Dr. Cronyn, after congratulating the author on the eloquent delivery and careful arrangement of the extracts presented from Louis, took exception to the doctrine of "typhoid affection," as sought to be proved thereby, quoting in corroboration of his opposition to the views of the author and of Louis—Budd, Chambers, Gardiner, Murchison, Tweedy, and others, and further that the antiphlogistic treatment which the author seemed to rely on as a part of the proof of the correctness of his Pyerian gland theory, had been most faithfully carried out by Andral, and *exactly* with corresponding fatality, concluding by insisting that notwithstanding post mortem examinations, such as Louis made, exhibited those glands in a diseased state; it was illogical to conclude therefrom that they were the primary seat of the morbific poison, but rather a secondary effect occurring in the natural process of elimination.

Dr. Lothrop said that he wished to compliment Dr. Strong upon his paper. He thought that in its manner it was admirable, in fact a model of research and style. With the matter of his paper he could not agree. In the first place he could not admit that all diseases involved some structure, so that diseased structure was at the foundation of all diseases. Upon this theory it would be hard to explain the important class of blood diseases, in which no structure was involved, unless it was assumed that the blood globule was such.

If M. Louis' doctrine, that the essential element of typhoid fever is the local affection of Peyer's patches, is true; if it is the primary affection and all else secondary, then Dr. Strong was not only right, but logical, in the treatment he recommended. But even assuming that local treatment of the inflamed patches by blistering may be the best course, the cases which Dr, S. had related were too few to prove anything. According to his statement they arrived at the crisis in about seven days, which was ordinarily reckoned one of the critical periods of the disease.

He believed, however, that the affection of the Peyerian patches was secondary. It was a constant lesion; Louis found it in all cases which he examined after death. The best modern authorities consider it secondary, the primary affection being a blood poison. Some think that the affection of the glands is in some way concerned in the elimination of the blood poison, or in other words that the special material in the blood for which the fever poison has an affinity, is eliminated through these glands, and hence the morbid changes in them.

In regard to the abreviation of the disease, it was probable that more could be affected in the early stages than was generally admitted. We know that if two persons exposed equally to the morbid fever poison, and each equally exhibiting the initiatory symptoms, viz: lassitude, headache, etc., one will go on with a regular course of the fever while the other will escape. We say in explanation that from the outset, though both appeared alike, one was to have a regular fever and the other not. This is probably not a good explanation. It is not right to say that the poison of typhoid is an entity, and if a man gets it he gets the whole, and its effects must go on; that he must either get the whole or none. The probability is that the effects are proportioned to the intensity of the poison. The more intense, the more severe will be its effects. If a man gets only a certain amount of the poison, nature may be competent to eliminate it before it has time to work its disorganizing influence upon the blood. The influence will then end with the initiatory symptoms. There is no doubt that by treatment we may aid nature in the elimination, and thus in a certain sense avert an attack of fever. If the poison is too intense to be eliminated by nature, aided or unaided, it will exhibit its influence in a regular course of fever, going on to crisis, or complete elimination.

But even under these circumstances it is a question whether its course cannot be abbreviated by treatment. Dr. James Jackson, whose opinions are always to be respected, made a number of observations to prove the effect of emetics given early, to shorten the course of typhoid, and if statistics are worth anything, proved that they shortened its duration. The same was true of cathartics. In other words means which are ordinarily called eliminative had a favorable influence upon the duration of the disease. It was an old belief that fevers could be broken up, and eliminatives, by skin, bowels, and kidneys, were used for that purpose. Perhaps the idea was not as absurd as it has of late been pronounced. There may be more in it than has been granted.

Dr. H. M. Congar was elected to read an essay at the next meeting.

Adjourned. T. M. Johnson, M. D., Sec'y.

